# ELECTIONS MATTER: AGING POLITICS AND POLICY IN THE BIDEN ERA

**DOI:** 10.1093/geroni/igac059.133

**Published:** 2022-12-20

**Authors:** Edward Miller, Janelle Fassi, Marc Cohen

## Abstract

The 2020 election of Joe Biden to the presidency has shifted the foundations of the American political system after four years of conservative policy leadership. Combined with a slim Democratic majority in Congress, the policies, priorities, and proposals of the Biden administration have important ramifications for all spheres of American life. Nowhere is this clearer than with older Americans who disproportionately supported Donald Trump’s reelection but are among those who benefit most with enactment of the Democratic party agenda. Between 2010 and 2050 the proportion of the U.S. population aged 65 years or older is projected to increase from 13% to 20%. Population aging creates opportunities and challenges for older adults, their families, and society in general. The Biden administration has chosen to meet these challenges, in part, by seeking to expand the social safety net. By contrast, the Trump administration largely supported Republican priorities to draw back on the federal government’s commitment to programs intended to support an aging population. This panel examines aging politics and policy during the first two years of the Biden administration. It reports on older adults’ voting patterns and the role of issue framing in limiting public support for proposals such as Medicare for All. It analyzes provisions in the American Rescue Plan Act and Build Back Better Bill to bolster formal care and services for older adults, as well as federal actions to support family caregivers. Edward Miller serves as panel chair; Janelle Fassi serves as co-chair and Marc Cohen as discussant.

